# Adverse Perinatal Outcome in Subsequent Pregnancy after Stillbirth by Placental Vascular Disorders

**DOI:** 10.1371/journal.pone.0155761

**Published:** 2016-05-26

**Authors:** Francesca Monari, Giulia Pedrielli, Patrizia Vergani, Elisa Pozzi, Federico Mecacci, Caterina Serena, Isabella Neri, Fabio Facchinetti

**Affiliations:** 1 Obstetric Unit, Mother Infant Department, University of Modena and Reggio Emilia, Modena, Italy; 2 Department of Obstetrics and Gynecology, University of Milano-Bicocca, Monza, Italy; 3 Department of Gynecology, Perinatology and Human Reproduction, University of Florence, Florence, Italy; Hospital de Especialidades del Niño y la Mujer de Queretaro, MEXICO

## Abstract

**Objective:**

To evaluate outcome in the pregnancy following a stillbirth (SB) by a placental vascular disorders.

**Study Design:**

A prospective, observational, multicenter study was conducted in woman with a history of stillbirth (> 22 weeks) between 2005 and June 2013, in 3 Italian University Hospitals. Causes of SB were previously identified after extensive investigations. Pregnant women were enrolled within the first trimester. The main outcome was “adverse neonatal outcome”, including perinatal death, fetal growth restriction, early preterm birth <33+6 weeks, hypoxic-ischemic encephalopathy, intracranial hemorrhage or respiratory distress.

**Results:**

Out of 364 index pregnancies, 320 women (87.9%) had a subsequent pregnancy during the study period. Forty-seven had an early pregnancy loss. Out of 273 babies, 67 (24.5%) had an adverse perinatal outcome, including 1 SB and 1 early neonatal death (3.7/1000).

Women who had a SB related to placental vascular disorders (39.6%), were at higher risk of an adverse neonatal outcome compared with women whose SB was unexplained or resulted from other causes (Adj. OR = 2.1, 95%CI: 1.2–3.8). Moreover, also obesity independently predicts an adverse perinatal outcome (Adj OR = 2.1, 95%CI: 1.1–4.3).

**Conclusion:**

When previous SB is related to placental vascular disorders there is a high risk for adverse neonatal outcomes in the subsequent pregnancy. Maternal obesity is an additional risk factor.

## Background and Objective

Although stillbirth (SB) is one of the most common adverse events in pregnancy in Western countries, there is a paucity of information regarding the management and outcome of the subsequent pregnancy [1]. Thus, one great challenge is how to best counsel families regarding their recurrence risk of SB and the optimal management of a subsequent pregnancy.

Several case series and meta-analyses reported a 2 to 10-fold increase in the risk of SB in women with a previous fetal demise compared to the general population [2–4].

Apart from recurrence, a history of SB was also associated with an increased rate of complications (placental abruption, preterm delivery, low birth-weight, and preeclampsia) in the subsequent pregnancy [5, 6]. Small studies reported that adverse perinatal outcomes are increased in women where previous SB was related to a “placental insufficiency” [7–10].

On the opposite, the risk of recurrence was similar to the general population in those with SB previously classified as “unexplained” [7, 11, 12]

Moreover, the management of pregnancy after SB include an increased rate of cesarean section and induction of labor, either because of medical concerns as well as due to patient and/or provider anxiety.

The aim of this study is to look at adverse perinatal outcome in pregnancies after SB by placental insufficiency compared to other causes of previous SB.

## Materials and Methods

We conducted a prospective, observational, multicenter study that involved three University Hospitals in Northern Italy (Modena, Monza and Firenze) who managed both high-and low-risk-population. The total number of deliveries at the 3 centers during the study period was 62730.

All cases of fetal death after 22 weeks of gestation were registered in a perinatal database, and each case of SB underwent the same work-up. This included a careful collection of information on obstetric history, the circumstances and causes of the SB, placenta histology, stillborn autopsy, microbiological evaluation, chromosome analysis, and neonate inspection by a neonatologist. Most relevant cause of death and associated conditions were audited by a multidisciplinary team (obstetrician, pathologist, neonatologist, and microbiologist) according to the CoDAC classification [13]. A comparison of different classifications demonstrated that CoDAC performs better in term of retaining important information and ease of use, reporting also a low proportion of unexplained cases [14–15].

In the period January 2005-June 2013, every woman with a history of SB who presented at the above centers during her first subsequent pregnancy was enrolled, before 12 weeks of gestation. All women lived in Northern Italy and had full access to medical care. For each woman, the obstetric history and lifestyle were assessed. A pre-pregnancy body mass index (BMI) ≥30 kg/m2 was defined as obesity. Information regarding prenatal care, invasive tests, prophylaxis and/or treatment during pregnancy was also collected. Gestational age at delivery, mode of delivery, complications and neonatal outcomes were recorded. Birth-weight centiles were calculated with customized rather than population curves [16]; this means that maternal age, pre-pregnancy BMI, parity and ethnicity were considered in addition to gestational age and gender. Fetal growth restriction was defined as a birth-weight below the 10^th^ centile according to customized curves. Miscarriage <12 weeks of gestation defined an early pregnancy loss.

For comparison, we collected data from the general population of parous women who delivered a live born baby at the 3 hospitals, during the study period.

The primary outcome was a composite “adverse neonatal outcome”, which included perinatal death, fetal growth restriction, early preterm birth (≤33^+6^ weeks), hypoxic-ischemic encephalopathy, intracranial hemorrhage or respiratory distress requiring ventilation. Pregnancy with prior SB due to placental vascular disorders (abruption, infarcts, insufficiency, Antiphospholipid Antibodies Syndrome, fetal growth restriction, severe preeclampsia) were compared to remnant SB, either unexplained or due to other causes. Secondary outcomes included separately the prevalence of SB/perinatal death, the rate of fetal growth restriction, low birth weight, preterm delivery (<37 weeks), and cesarean section.

Data were analyzed using Statistical Package for the Social Sciences Version 20.0 (SPSS Inc., Chicago, IL, USA). Continuous variables with normal distribution are expressed as means ± standard deviation, and categorical variables are expressed as frequencies and percentages. Odd Risk (OR) with 95% confidence interval (CI) was computed when appropriate.

Study was approved by Ethics Committees of the Universities of Modena, Firenze and Monza. All women provided written informed consent.

## Results

The cohort included 364 women with a history of ante-partum SB (index pregnancy) of which 322 women became pregnant during the observation period (subsequent pregnancy). The mean gestational age at enrollment was 7^+5^ weeks (range: 4–12 weeks).

**Fig 1:**
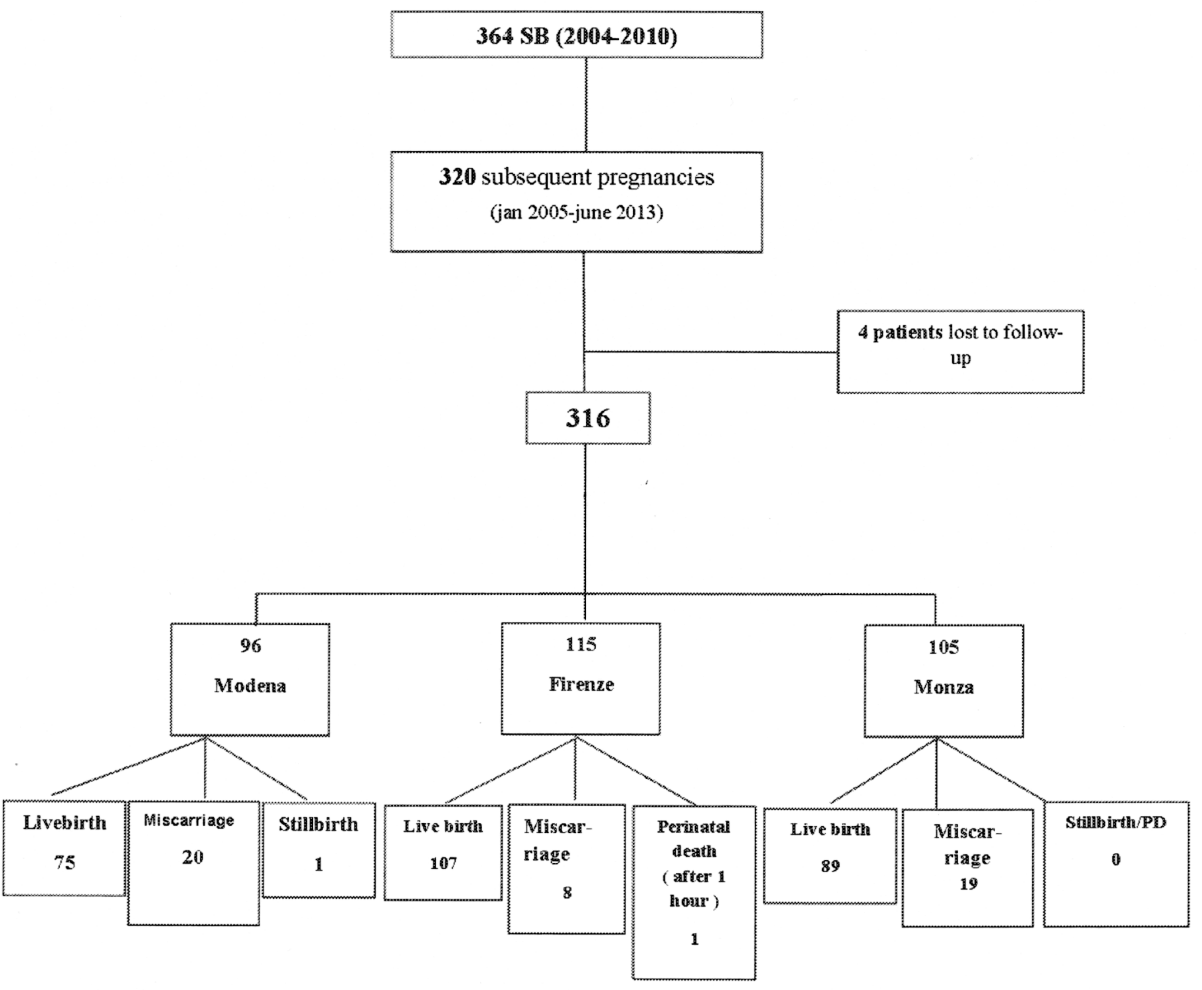
The flow chart of the study.

One woman had a twin pregnancy, and another one had quadruplets. Four women were lost to follow up. Thus, the complete maternal and neonatal outcomes were obtained in 320 pregnancies giving birth to 273 babies. An early pregnancy loss occurred in 47 women (14.7%, Fig 1).

Out of 273 neonates, one was stillborn because of placental abruption (3.7/1000) and 1 died at first hour of life because of asphyxia (3.7/1000). In the same period, the rate of SB and early neonatal death in the general population was 4.9/1000 and 2.6/1000, respectively.

In the subsequent pregnancy, 37.8% of the mothers had an age >35 years. For this reason, 37.2% of the women received a genetic amniocentesis. Obesity rate increased from 10.0 to 15.6% (OR = 1.67; 95%CI: 1.0–2.7) compared with the index pregnancy. Moreover, a lower percentage of women received <4 antenatal visits (9/320 vs 125/320; OR = 0.05; 95%CI: 0.0–0.1) and <2 ultrasound examinations (5/320 vs 131/320; OR = 0.02; 95%CI: 0.0–0.1) compared with their index pregnancy.

All women had been screened for thrombophilia after SB, and 40 (14.9%) tested positive, which included 35 for congenital thrombophilia (factor V Leiden or factor II G20210A mutation) and 5 women for acquired thrombophilia (anti-cardiolipin antibodies and lupus anticoagulants). These latter received both low-dose aspirin plus low-molecular weight heparin (LMWH) prophylaxis.

The frequency of cases with SB related to placenta vascular disorders was 39.6% (108/273) while in 16.1% SB was related to other causes (44/273). In the remnant 44.3% SB was unexplained (Table 1). The composite adverse neonatal outcome occurred in 67 cases (24.5%), equally distributed in the 3 centers (Fig 1). Adverse neonatal outcome were more prevalent in cases with a previous placenta vascular disorders SB (32.4%) compared with cases where SB was related to other causes or unexplained (19.4%) (35/108 vs 32/165; OR: 2.00, 95%CI: 1.14–3.48, p = 0.015) (Table 1).

**Table 1:** Adverse neonatal outcome in relation to cause of previous SB.

Previous cause of death *	Adverse neonatal outcomes 67	Uneventful pregnancy 206	OR (95% C.I.)*
**PVD (108)**	**35 (52.2)+**	**73 (35.4)**	**1.96 (1.1–3.5)**
Abruptio (12)			
Infarcts/insufficiency (39)			
Antiphospholipid Antibodies Syndrome (3)			
IUGR (51)			
Severe preeclampsia (3)			
**Other (44)**	**11 (16.4)**	**33 (16)**	**1.03 (0.5–2.2)**
Infections (14)			
Congenital anomalies (16)			
Diabetes (2)			
Extreme prematurity/cerebral hemorrhage (5)			
Hydrops (7)			
Twin to twin transfusion syndrome (0)			
**Unexplained (121)**	**21 (31.3)**	**100 (48.5)**	**0.48 (0.3–0.9)**

* Specific causes/ associated conditions of previous SB are reported in brackets.

+ Numbers with % in brackets.

At univariate analysis, other factors were associated with adverse neonatal outcome, such as obesity (17/67 vs 28/206, *p =* 0.038), LMWH prophylaxis (38/67 vs 85/206, *p =* 0.039) and a trend with smoking (9/67 vs 16/206, *p =* 0.16). At multivariate regression, adverse neonatal outcome was predicted by maternal obesity and SB related to placental vascular disorders (*chi-squared*: 10.45, *p =* 0.03; *R squared*: 0.038) Table 2.

**Table 2:** Factors associated with adverse neonatal outcome or FGR (univariate analysis and logistic regression).

	OR (95% C.I.)	Adjusted OR (95% C.I.)	p =
**Adverse neonatal outcome**			
Factors*			
Obesity	2.16 (1.0–4.5)	**2.1 (1.1–4.3)**	**0.031**
Previous SB related to PVD	1.96 (1.1–3.5)	**2.1 (1.2–3.8)**	**0.046**
Smoking	1.9 (0.9–4.7)		
Previous Unexplained SB	NS		
Age >35 years	NS		
Thrombophilia	NS		
LMWH prophylaxis	2.1 (1.2–3.9)	**-**	
Ultrasounds < 2 exams	NS		
Antenatal visits < 4	NS		
**Fetal Growth Restriction**			
Factors*			
Obesity	2.0 (1.0–4.2)	**2.59 (1.21–5.5)**	**0.009**
Previous SB related to PVD	2.3 (1.2–4.2)	**-**	
Smoking	2.6 (1.1–6.4)	**-**	
Previous Unexplained SB	NS		
Age >35 years	NS		
Thrombophilia	NS		
LMWH prophylaxis	2.3 (1.2–4.4)	**-**	
Ultrasounds < 2	NS		
Antenatal visits < 4	NS		

Women with SB related to placenta vascular disorders were at high risk for very low birth weight (OR: 3.65, 95%CI: 1.09–12.19, p = 0.03), preterm birth (OR: 1.95, 95%CI: 1.06–3.61, p = 0.03) and cesarean section (OR: 1.80, 95%CI: 1.09–2.97, p = 0.02) respect with women where SB was related to other causes/ unexplained.

At univariate analysis, fetal growth restriction was associated with obesity (16/56 vs 29/217, p = 0.011), SB related to placenta vascular disorders (29/56 vs 79/217, *p = 0*.*03*), smoking (9/56 vs 16/217, *p =* 0.04) and LMWH prophylaxis (34/56 vs 9/217, *p =* 0.01). At multivariate regression only maternal obesity predicted fetal growth restriction (*chi-squared*: 13.20, p = 0.01; *R squared*: 0.047) Table 2.

The case of recurrent SB occurred at 31+5 weeks in a young overweight Indian woman with congenital thrombophilia (factor II G20210A mutation) because of placental abruption. The stillborn had recurrent severe fetal growth restriction (< 3^rd^ centile). Patient had received 9 antenatal visits and 5 ultrasound examinations with Doppler velocimetry. Her previous SB had occurred at 28 weeks. The case of neonatal death occurred in a young healthy Italian woman at 39 weeks gestation. After an emergency cesarean section because of an abnormal non-stress test, she delivered a live born baby with severe growth restriction (< 3^rd^ centile) and a 5-minute Apgar score of 3. Despite resuscitation, the baby died 1 hour after birth. She had received amniocentesis, 7 antenatal visits and 5 ultrasound examinations with Doppler velocimetry. Previous SB had occurred at term. In both cases, the previous SB was classified as the result of placental vascular disorders. Both women had received LMWH prophylaxis.

Table 3 compares the outcomes in the subsequent pregnancies and those in the general population of multiparous women attending the 3 hospitals. The rate of preterm birth (<37 weeks) was significantly higher in the subsequent pregnancies (18.7% versus 9.2%, OR = 2.4, 95%CI: 1.7–3.2) than in the general population. The same occurred for low birth weight babies (17.9% vs 9.2%, OR = 1.8, 95%CI: 1.3–2.4). Moreover, the rate of cesarean section (37.1% vs 24.7%, OR = 1.8, 95%CI: 1.5–2.3) and induction of labor (35.7% vs 17.3%, OR = 1.8, 95%CI: 1.4–2.3) were higher than in the general population.

**Table 3:** Outcomes of NP (excluding early pregnancy loss) compared with multiparous general population.

	Subsequent pregnancy/neonates 269/273	Multiparous population 32,568	OR (95%CI)
**Gestational age**			
< 33+6 weeks (<237d)	18 (6.7%)	1475 (4.5%)	1.5 (0.93–2.45)
34–36+6 weeks (238-258d)	33 (12.3%)	1523 (4.7%)	2.8 (2.0–4.1)
> 37 weeks (>259d)	218 (81%)	29570 (90.8%)	0.4 (0.3–0.6)
**Mode of delivery**			
Spontaneous labor	108 (39.7%)	20060 (61.6%)	0.3 (0.3–0.4)
Emergency CS	11 (11.4%)	1025 (5.1%)	2.4 (1.3–4.5)
Induction of labor	96 (36.1%)	5640 (17.3%)	2.6 (2.1–3.4)
Emergency CS	24 (25%)	1185 (21%)	2.6 (1.7–4)
Elective CS	65(24.2%)	5840 (17.9%)	1.5 (1.1–1.9)
**Birth- weight (grams)**			
< 1500	13/237(4.8%)	732 (2.2%)	2. 5 (1.4–4.4)
1501–2500	34/273 (12.4%)	2272 (7.0%)	1.9 (1.3–2.7)
> 2501	226/273(82.8%)	29564 (90.8%)	0.5 (0.4–0.7)

Thrombophilic women were more likely to receive LMWH prophylaxis compared with those tested negative (31/40 vs 92/228, OR = 5.1; 95%CI: 2.3–11.2). In the subset of thrombophilic women, the outcomes were unrelated to LMWH prophylaxis with the exception of an increased risk for low-birth-weight babies (13/31 vs 8/9; OR = 13.2; 95%CI: 5.0–34.8). Composite adverse neonatal outcomes were not different in trombophilic and not trombophilic women (13/40 vs 48/229, OR = 1.8; 95%CI: 0.9–3.8).

## Comment

Our findings confirm that the subsequent pregnancy after SB is associated with an increased risk of adverse outcomes (fetal growth restriction, preterm birth, etc), compared with the general multiparous population [17]. However, perinatal outcomes differ according to the cause of SB in the index pregnancy. Indeed, they were more frequent when SB was related to a placental vascular disorder. Anecdotally, also the two cases of recurrent perinatal death we observed occurred in women with prior SB associated with placental insufficiency.

This finding indicates an intrinsic risk of recurrence in such cases, as also suggested by histological observations [18]. A support is given by Gordon et al., who demonstrated that a delivery of a small for gestational age baby in the first pregnancy is associated with an increased risk of SB in the second one [19]. Recently, smaller studies demonstrated that prior placental SB is associated with fetal growth restriction and other complications in subsequent pregnancies [8, 9]. A large population-based study from Australia [16] indicated that delivering a small for gestational age neonate in the first pregnancy increases the risk of SB in the next one, especially in premature birth. Moreover, women who experienced SB in their first pregnancy were more likely to have infant mortality in subsequent ones [20].

All together, the above observations and our findings put forward the concept that a history of SB predicts increased risk for adverse perinatal outcomes in future pregnancies only if a placental insufficiency characterized the previous one. Indeed, when SB is related to a different cause, the occurrence of adverse perinatal outcome in the subsequent pregnancy is low. The logical consequence is the need to correctly classify each and every SB with thorough exams, (a procedure seldom applied), in order to counsel couples, as already suggested [10].

In addition to a positive history for placental vascular disorders, an adverse neonatal outcome was independently predicted by maternal obesity, which represents a well-documented risk factor for SB [21– 22], while no correlation was found with a short inter-pregnancy interval (less than 6 months), as already reported [23].

Overall, women with prior SB received a considerable amount of medical care in their subsequent pregnancy with more invasive procedures, ultrasound exams and antenatal visits compared with index pregnancy. Nonetheless, the prevalence of obesity increased by 50% in our cohort, while the rate of smoking remained stable. Thus, it is clear that our physicians did not effectively counsel women on how to reduce their SB risk, rather they increased rate of labor induction and elective cesarean section (indicated just for “previous SB”) as already reported in the U.S. and Canada [3, 11]. As expected, thrombophilia was highly prevalent in the group of women with SB [24]. Although prophylaxis with LMWH proven to be without benefit [25], even independently of thrombophilic status we observed an overtreatment with LMWH. Therefore, all the above interventions could be explained by patient/provider anxiety feelings.

The strengths of this study include its prospective design, early enrollment and a complete diagnostic evaluation of the causes and associated conditions of the previous SB. This cohort is one of the few with extensive details and prospective ascertainment of patients. The study limitations are the unknown rate of early pregnancy loss in the general population, which restricted the investigation of this negative outcome and the small sample size, which cannot allow evaluation of the rate of SB recurrence.

In conclusion, the subsequent pregnancy after SB is associated with an increased risk of adverse perinatal outcomes, only in case SB was associated with placental vascular disorders. Maternal obesity represents an additional risk factor. Efforts should be made to both identify causes of SB and reduce modifiable risk factors, such as obesity and smoking.

## Supporting Information

S1 Data (XLS)
